# Pomegranate Extracts in the Management of Men's Urologic Health: Scientific Rationale and Preclinical and Clinical Data

**DOI:** 10.1155/2013/701434

**Published:** 2013-03-26

**Authors:** Kroeger N., Belldegrun A. S., Pantuck A. J.

**Affiliations:** ^1^Department of Urology, Institute of Urologic Oncology, David Geffen School of Medicine at the University of California, 924 Westwood Boulevard, Suite 1050, Los Angeles, CA 90095-7384, USA; ^2^Department of Urology, University Medicine Greifswald, F.-Sauerbruch-Str., 17 489 Greifswald, Germany

## Abstract

Multiple strands of research provide growing evidence that diet, nutrition, and life style play a role in the development and the course of urological diseases. Numerous micronutrients and polyphenols found in soy, green tea, and many fruits and vegetables have been described to impact diseases including erectile dysfunction, benign prostatic hyperplasia, and prostate cancer. However, oftentimes these reports lack both a scientific rationale and supportive evidence base. The efficacy of pomegranate, on the other hand, in the modulation of central biological processes like inflammation, hypoxia, and oxidative stress that are important in the pathogenesis of urological maladies has been robustly demonstrated in preclinical *in vitro* and *in vivo* studies. Moreover, clinical trials have further supported its use in the treatment of several diseases, in particular in the management of prostate cancer. Herein, we critically review the scientific knowledge about the current role and future prospects for the use of pomegranate extracts in the therapy of erectile dysfunction, benign prostatic hyperplasia, and prostate cancer.

## 1. Introduction

As men age, there are inevitable changes that occur in the genitourinary tract that increase their risk of developing urological diseases such as ED (erectile dysfunction), prostatitis, BPH (benign prostatic hyperplasia), and PCA (prostate cancer). An autopsy study reported an incidence of BPH of only 8 percent in men during the fourth decade of life, while 50 percent of men have pathological BPH between ages 51 and 60 [1]. Likewise, data from the Massachusetts Male Aging Study (MMAS) have demonstrated an increase in the incidence of ED with each decade of age, rising from 12.4 cases per 1000 men years (95% CI 9.0 to 16.9) in men aged 40–49 to 46.4 cases per 1000 men years (95% CI 36.9 to 58.4) in men aged 60–69 years [2].

In the past years, there is a growing body of literature that supports the role of environmental and other life style factors in impacting pathological alterations of the genitourinary tract. For example, Asian men usually have a significantly lower incidence of and mortality from prostate cancer when compared with men in Western Europe and North America. These differences frequently are mitigated after immigration to Western countries after only one generation [3]. Authors have suggested that this finding is a result of westernization, and adoption of western lifestyle and nutrition habits [4]. The increase in incidence of prostate cancer in Asian countries [5] along with a growing economy in these countries supports the hypothesis that there are both environmental and genetic influences which impact the development of prostate cancer.

Modifiable lifestyle and nutrition habits and the long latency of many problems of men's health make them ideal candidates for prevention as well as complementary treatment strategies. Moreover, urological patients have a high desire to get alternative and complementary treatments. A meta-analysis has demonstrated that 30% of prostate cancer patients are using complementary alternative medicine (CAM) in addition to their standard treatments [6]. In search for new treatment and prevention approaches, research has suggested that reduction of oxidation and inflammation may improve health outcomes. Increasing evidence suggests that some of these risks appear to be reduced by nutritional interventions that may help support the maintenance of men's health in general and the lower urinary tract in particular (reviewed in [7]).

Current dietary recommendations emphasize increasing the daily consumption of fruits and vegetables from diverse sources such as citrus fruits, cruciferous vegetables, and green and yellow vegetables. There are potent phytochemicals in edible plants, which may have health benefits through antioxidation, inhibition of inflammation, and via gene-nutrient interactions. In order to explain preventive as well as therapeutic effects of nutrition components, investigators have focused on the antioxidative effects of plant polyphenols [7]. Extracts derived from pomegranate (*Punica granatum*) have been intently studied over the past decade. Preclinical and clinical studies provide evidence of their antiproliferative properties and modulatory effects on inflammatory pathways [8]. Herein, we will provide an overview on the current preclinical and clinical knowledge of the mode of action and the use of pomegranate in the prevention and treatment of ED, BPH, and PCA.

## 2. Biological Rational for the Use of Pomegranate Extracts in the Treatment of Urological Diseases

Pomegranate phytochemicals have been shown to be effective in reducing oxidative stress and to modulate inflammatory pathways [9, 10]. The rationale for the use of pomegranate in prevention and treatment of ED, BPH, and PCA, therefore, depends on the influence of inflammation and hypoxia on the pathogenesis of these diseases. 

BPH is a urological disease that is characterized histologically by glandular hyperplasia in the periurethral and transition zones of the prostate, and clinically by obstructive and irritative lower urinary tract symptoms (LUTS) [11]. Accumulating evidence suggests that inflammation may be involved in the development of BPH/LUTS [12]. In rodent models, induced prostatic inflammation has been shown to contribute to prostatic hyperplasia [13, 14], and in humans, inflammatory infiltrates are frequently observed in prostate tissue specimens from men with BPH [15, 16]. Moreover, the presence or degree of inflammation has been found to be correlated with prostate size [16, 17], severity of LUTS [18], and greater risk of developing acute urinary retention [19]. Given these observations suggesting that inflammation may contribute to BPH/LUTS, it is plausible to hypothesize that anti-inflammatory agents may reduce the severity of lower urinary tract symptoms associated with BPH.

In 1863, Rudolf Virchow firstly made a link between inflammation and cancer when he noted “lymphoreticular infiltrates” within neoplastic lesions. It has been estimated that about 15% of cancers may be attributed to infectious agents and to the concomitant inflammation that is a major component of chronic infections [20]. Balkwill and Mantovani have labeled genetic damage as the “match that lights the fire” and inflammation as the “fuel that feeds the flames” [20]. Chronic inflammation is common in the prostate of men with advanced ages and can be found in approximately 80–98% of prostate biopsies [21, 22]. Evidence supports the role of proliferative inflammatory atrophy as a precursor to prostatic intraepithelial neoplasia and invasive prostate cancer [23]. The risk of prostate cancer may be reduced with the prophylactic intake of anti-inflammatory drugs. Meta-analyses, for example, have shown a ~15–20% risk reduction in patients who are using anti-inflammatory pharmaceuticals such as nonsteroidal anti-inflammatory drugs (NSAIDs) or aspirin on a regular basis [3, 24, 25]. However, the chronic use of NSAIDs and aspirin is limited by its associated side effects, including gastric ulcers, renal insufficiency, and in the case of COX-2 inhibitors, with cardiovascular events (reviewed in [26]). Prostate cancer is a slow-growing disease and, therefore, it will be mandatory to find prevention strategies which are effective in decreasing inflammation without causing side effects when used in the long term.

Inflammation is inextricably linked with hypoxia and an occurrence of reactive oxygen species (ROS). Evidence supports that inflammation and secondary ROS play important roles in prostate carcinogenesis [27]. An imbalance in the intracellular production and clearance of ROS results in oxidative stress, which in turn leads to an increased risk of DNA damage and other epigenetic changes [28]. As in other cancer types, oxidative stress, low levels of antioxidative enzymes, and defective DNA repair of oxidative DNA damage have been implicated in prostate carcinogenesis [3, 29].

**Figure 1:**
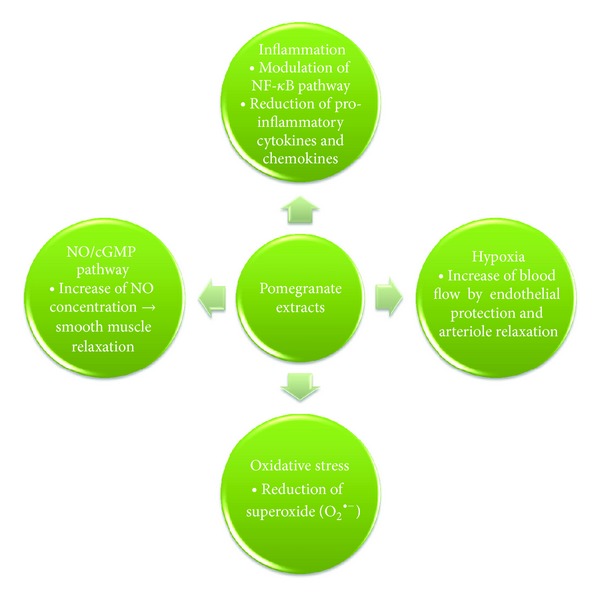
Potential modulatory effects of pomegranate extracts on central biological processes that have a crucial role in the pathogenesis of erectile dysfunction (ED), benign prostatic hyperplasia (BPH), and prostate cancer (PCA).

In the past few years, it has become increasingly apparent that the nitric oxide (NO)/cyclic guanosine monophosphate (cGMP) pathway has a major influence on the pathogenesis of both LUTS and ED. LUTS is one of the most important risk factors for the development of ED. A study of Germany has shown that 72.2% of men with ED had LUTS compared to only 37.7% without ED. In a multiple logistic regression analysis, age, diabetes, hypertension, pelvic surgery, and LUTS were all independent risk factors for the development of ED [30]. In both conditions, an increase of smooth muscle tension is thought to play a central role in the pathophysiology. The NO/cGMP pathway is one of the major regulators of smooth muscle contractility of the lower urinary tract and the penile corpora cavernosum and spongiosum. NO is a neurotransmitter of the vegetative nervous system. It is released, that is, by sexual stimulation from nonadrenergic, noncholinergic (NANC) neurons that innervate the corpus cavernosum of the penis [31]. The expression of NO is dependent on the presence of oxygen and reduced nicotinamide-adenine dinucleotide phosphate (NADPH) and is catalyzed by the NO synthase (NOS). NO activates the intracellular guanylate cyclase, the enzyme that further controls the conversion of 5-GTP to 3′, 5′-cGMP. The accumulation of intracellular cGMP triggers a cascade, leading to decreased intracellular calcium levels and subsequent relaxation of smooth muscles of various organs, including the arteries and the lower urinary tract [32–34]. BPH and ED are both diseases that become increasingly prevalent as patients age, and in consequence, are often accompanied with disorders that also occur more frequently in advanced ages like diabetes mellitus, hypertension, arteriosclerosis, and hypercholestrolemia. All of these covariates have been well described as risk factors of ED. Moreover, it has been demonstrated that these factors are associated with an increase in the intracavernosal expression/activity of reduced NADPH-oxidase, an enzyme that generates superoxide (O_2_^•−^) [35]. O_2_^•−^ is a highly reactive radical that can interrupt the physiological function of NO due to its ability to react with NO to form peroxynitrite (PN). NO as well as PN is able to mediate smooth muscle relaxation of the corpus cavernosum, but PN is much less potent compared to NO [36]. Recent phase III studies demonstrating the efficacy of phosphodiesterase (PDE) inhibitors in the treatment of LUTS and ED [37–40] have highlighted the crucial role of a functional intact NO/cGMP pathway for both urological disorders. On overview of potential effects of pomogranate on diverse biological mechanisms is shown in Figure 1.

## 3. Preclinical Evidence for the Use of Pomegranate Extracts in Men's Urologic Health

### 3.1. Prostate Cancer

The most striking evidence for the biological activity of pomegranate extracts was demonstrated in prostate cancer cells. *In vivo* and *in vitro* studies have shown that pomegranate extracts are capable of inhibiting tumor cell proliferation, migration, and invasion, while also inducing apoptosis. For example, only 70 microg/mL of pomegranate extract was needed to suppress the growth of LNCaP prostate cancer cells by 50%, whereas normal prostate cells (hPrECs) were considerably less affected (ED_50_ 250 g/mL) [41]. Incubation of LNCaP cells over 24 h at various concentrations revealed that punicic acid, a fatty acid present in pomegranate seeds, was able to stimulate apoptosis in a caspase-dependent manner [42]. Koyama et al. [43] examined the relationship between POMx- (POM Wonderful LLC, Los Angeles, CA, USA), a pomegranate extract with standardized ellagitannin content (37% punicalagins by HPLC), induced apoptosis and the insulin growth factor (IGF)/insulin growth factor binding protein- (IGFBP-)3 system in LAPC4 prostate cancer cell lines. Cultivation with POMx and IGFBP-3 had additive effects on apoptosis induction and growth inhibition. Western blot analyses of the proapoptotic c-Jun kinase (JNK) and the growth inductors of the mTOR pathway indicated a downregulation of the mTOR pathway and an increase in JNK expression. 

Moreover, in addition to causing prostate cancer cell death, pomegranate extracts have been reported to increase cell adhesion and decrease cell migration, processes important for metastatic spread. For example, treatment of prostate cancer with pomegranate extracts resulted in an upregulation of genes involved in cell adhesion (E-cadherin, intercellular adhesion molecule 1 (ICAM-1)) while genes that are important for cell migration (hyaluronan-mediated motility receptor (HMMR) and type I collagen) were downregulated. Furthermore, cytokines that are known mediators of inflammation (IL-6, IL-12p40, and IL-1*β*) and are capable of chemoattracting cancer cells via the SDF/CXCR4 axis were downregulated by pomegranate extracts [44]. 

Our institution has demonstrated the inhibitory effects of pomegranate extracts on inflammatory pathways [10]. The nuclear factor-kappaB (NF-*κ*B) is an important predictor of prostate cancer biochemical recurrence [45, 46] and is activated in castration-resistant prostate cancer (CRPC) [47]. Our investigations have demonstrated that inhibition of NF-*κ*B is necessary for the maximal proapoptotic effect of pomegranate extracts. Moreover, these studies also showed that pomegranate extracts not only inhibit the activation of NF-*κ*B, but also delay the emergence of castration resistance in a xenograft model through reduced proliferation and increased apoptosis. These results were further supported by a recent study that comparatively analyzed proteomic patterns of treated and untreated DU145 cells [48]. After treatment with pomegranate extracts, 11 proteins were deregulated in treated DU145 cells. Three proteins were upregulated and eight proteins were downregulated. Among the proteins that were downregulated after treatment with pomegranate was valosin containing protein (VCP (p97)). Elevated expression of VCP is known to be a prognostic factor for tumor progression and metastasis of PCA [49]. Of note, VCP belongs to the ATPase superfamily and is involved in the ubiquitin/proteasome-mediated degradation of I*κ*B-*α*, the endogenous inhibitor of NF-*κ*B. Therefore, VCP is capable of regulating the activation of NF-*κ*B [50]. Thus, the decreased expression of VCP could be one mechanism that may explain the NF-*κ*B-inhibitory effect and consequently the proapoptotic and antiandrogen effects of pomegranate extracts.

Finally, remarkable results on the tumor growth inhibitory properties of pomegranate extracts were recently reported by Adhami and colleagues [51]. The transgenic adenocarcinoma of the mouse prostate (TRAMP) model is one of the most relevant models to humans because mice uniformly spontaneously develop orthotopic prostate tumors following the onset of puberty [52]. In their study, Adhami et al. [51] supplemented drinking water with 0.1% and 0.2% of pomegranate extracts, comparable to 250 to 500 mL of pomegranate juice. In the control group, 100% of mice developed palpable tumors after 20 weeks compared with only 30 and 20% in the 0.1 and 0.2% pomegranate extract-supplemented groups, respectively. The median life expectancy was 43, 73, and 92 weeks in the control, 0.1%, and 0.2% groups, respectively. Unlike the control group, none of the mice in the pomegranate-extract-treated groups developed metastases, and all tumors were without any evidence of poorly differentiated features. In concordance with previous *in vitro* studies, the authors reported a simultaneous and significant inhibition of the IGF-I/Akt/mTOR pathways in the PCA resulting from treatment with prostate extracts.

### 3.2. Erectile Dysfunction

In an effort to search for markers of oxidative stress in arteriogenic ED and to investigate the protective role of dietary antioxidants, Zhang et al. [53] developed atherosclerosis-induced ED in rabbits by balloon deendothelialization of the iliac arteries. Analogous to the situation in patients with atherosclerosis, they found a decreased erectile tissue blood flow, diminished intracavernosal perfusion pressures, and impaired metabolic waste clearance from the erectile tissues. Moreover, they detected an accumulation of oxidatively modified byproducts and an upregulation in levels of superoxide-dismutase and aldolase reductase, both of which are known to be oxidation-sensitive genes. Contrarily, in the pomegranate-extract-treated group animals had an increased intracavernosal blood flow, smooth muscle relaxation, and erectile activity compared to controls. However, the erectile activity of the pomegranate-treated group did not normalize to the level of age-matched animals without arteriosclerosis. The authors concluded from their study that antioxidative therapy appears to act promptly on molecular and ultrastructural alterations rather than on anatomical structures like erectile tissue fibrosis. Additionally, they suggested that long-term consumption of antioxidative dietary improves ED by protection of NO bioavailability. Similar results were reported by Azadzoi et al. [54] who also reported an increased intracavernosal blood flow, improvement of erectile response, and smooth muscle relaxation in an animal model. Furthermore, they found, opposite to Zhang et al., that long-term intake of pomegranate extracts also helped to prevent erectile tissue fibrosis in the treated ED group.

## 4. Clinical Evidence for the Efficacy of Pomegranate Extracts in Urological Diseases

### 4.1. Prostate Cancer

A number of clinical studies have provided evidence of biologic activity of pomegranate extracts in human prostate cancer. In a phase II study, the effects of daily consumption of 8 oz. of pomegranate juice on PSA progression in 46 men with a biochemical recurrence after surgery or radiation were assessed [55]. Eligible patients had a detectable PSA >0.2 and <5 ng/mL and s Gleason score ≤7. The clinical endpoints of the study were safety, effect on serum PSA, serum-induced *in vitro* proliferation and apoptosis induction of LNCaP cells, serum lipid peroxidation, and serum nitric oxide levels. During the study period, no serious adverse events were reported. There was a statistically significant prolongation of the PSA doubling time from a mean of 15 months at baseline to 54 months after treatment (*P* < 0.001). The effects of patient sera following pomegranate consumption on proliferation and apoptosis induction on LNCaP prostate cancer cells *in vitro* were measured. Proliferation assays demonstrated a mean decrease of proliferation by 12% (*P* = 0.0048) between sera at baseline and after 9 months of pomegranate consumption. In the same assay system, apoptosis increased by 17.5% (*P* = 0.0004) after cultivation with the same patient media. In addition, susceptibility against oxidative stress, measured by the content of serum lipid peroxides, significantly decreased following pomegranate juice consumption. This was the first clinical study to provide evidence beyond preclinical investigations of the biologic activity of pomegranate juice consumption on recurrent prostate cancer. 

Recently, Paller et al. [56] published a multicenter phase II clinical trial of men with localized prostate cancer to receive either 1 or 3 g of POMx pills. POMx is a standardized pomegranate (*Punica granatum* L., Wonderful variety) polyphenol extract developed for use as a dietary supplement and which has received “Generally Recognized as Safe” status. Each capsule contained up to 1000 mg of polyphenol extract, which delivers pomegranate polyphenols in an amount equivalent to approximately 8 oz of pomegranate juice. The extract is well characterized, product specifications have been established, and batch analyses data confirm that the product is consistent in quality and free of microbial or chemical contaminants. It contains the same compounds found in pomegranate juice, differing only in having lower anthocyanidins and significantly higher proportional content of pomegranate polyphenols, primarily punicalagin and isomers, but the levels in food or supplement products are limited to the amount found in 8 oz of 100% juice. The study cohort of 104 patients was stratified according to their baseline PSA doubling time (PSADT) and Gleason score. The primary endpoint of the study was a detection of a 6-month on-study increase in PSADT. PSADT lengthened from 11.9 months at baseline to 18.8 months after treatment in the intention to treat group (*P* < 0.001); however, there was no evidence of dose-response impact on PSADT between the two dosage groups (*P* = 0.554). A decline in PSA was observed in 13% of patients. Again, there were no severe side effects of the treatment; however, mild diarrhea was reported in 1.9% of the low-dose group and 13.5% in the high-dose group. This study confirmed previously reported findings, but in a more heterogeneous patient cohort (31% of patients in this study had PSA >5 ng/mL with up to 32 ng/mL). A major limitation of this study was the high dropout rate of 42% of patients before the protocol definition of PSA progression or the 18 months of followup were reached. The most important reason for premature discontinuation of the trial was uncertainty of the patient and/or the investigator in case of PSA progression. Collectively, 70% of the study population remained on study medication for 12 months, judged to be sufficient enough to provide an adequate patient number to reliably examine PSADT.

Major criticisms of these two phase II studies have been the lack of placebo control, the lack of a dose-response effect, and the lack of prostate tissue to correlate PSA change with *in vivo* biological effects. Moreover, the fact that a prior placebo-controlled trial found 73% of men on placebo on a similarly designed study had longer on-study PSADT than prestudy [57] makes interpreting these data challenging. Several clinical studies have been performed to address these deficiencies. A follow-up multicenter, phase III, double-blind, placebo controlled study of pomegranate juice extract (NCT00060086) in 180 men with biochemically recurrent prostate cancer has recently been completed, with the results anticipated in the first quarter of 2013. Additionally, a randomized, placebo-controlled clinical trial of POMx daily for up to 4 weeks prior to radical prostatectomy in order to obtain prostate tissue was conducted to objectively measure whether pomegranate extracts and their metabolites were systemically absorbed and accumulated in the prostate [58]. The primary study outcome was the difference between arms in prostate 8-hydroxydeoxyguanosine (8-OHdG) levels. 8OHdG is formed as the result of oxidative damage to the DNA base 2′-deoxyguanosine (dG) and is a major product of DNA oxidation. This study gave for the first time clinical evidence for the accumulation of pomegranate extracts in benign and malignant prostate tissues. Urolithin A (3, 8-dihydroxy-6*H*-dibenzo[*b*,*d*]pyran-6-one), a metabolite of ellagic acid, punicalagin, and the ellagitannin extract that are all ingredients of pomegranate, was more often detected (*P* = 0.031) with higher levels in the POMx arm compared to placebo-treated patients (*P* = 0.007). In both benign and malignant prostate tissues, there was an inverse correlation between Urolithin A and the levels of oxidative DNA damage as measured by 8-OHdG levels. POMx reduced benign prostate tissue 8-OHdG by 33% (*P* = 0.003) in men with organ-confined disease. This was (a) a modest size study and (b) the time of treatment was within 4 weeks very short. As discussed in the previous sections, it has been suggested that the effects of pomegranate extracts become evident in long-term use by its ability to reduce chronic oxidative stress. Thus, the most important finding of this study was the proof of metabolite accumulation in the prostate and the strong trend for its association with reduced 8-OHdG in both benign and malignant tissues.

Another point of criticism to the studies above is the use of PSADT as a surrogate parameter for standard clinical outcomes such as survival. A retrospective study presented at the ASCO Meeting 2012 has shown that PSADT may increase even in the absence of therapy, possibly in part due to the duration of PSA followup [59]. The findings of these investigations challenge the use of PSA kinetics in single-arm studies that do not have a placebo-controlled comparator. 

In summary, the current clinical trials provide ample evidence for the biological activity of pomegranate extracts in prostate cancer. This conclusion has to be emphasized in the light of other alternative treatments with agents like celecoxib or rosiglitazone that did not improve the mean PSADT when compared to a placebo control group in randomized clinical trials [57, 60].

### 4.2. Erectile Dysfunction

One study investigated the improvement of ED in 60 sexually active, healthy males aged 21–70 years [61]. Inclusion criteria included mild-to-moderate ED, as indicated (17–25 points of the International Index of Erectile Function (IIEF) questionnaire [62]), and a stable monogamous relationship with a consenting female partner. The study was designed as a crossover clinical trial. Patients were assigned to two groups that either received 8 oz of pomegranate juice or placebo for a total of 28 days. After a washout period of two weeks, the placebo group switched to the active treatment and vice versa. At the end of the second period, subjects were evaluated with the IIEF questionnaire again. An improvement in Global Assessment Questionnaire (GAQ) was the primary endpoint and a change of the erectile function domain in the IIEF questionnaire was the secondary endpoint. The authors observed a trend towards an improvement of GAQ associated with the consumption of pomegranate juice treatement (*P* = 0.058). The secondary efficacy endpoint, an upgrading of the erectile function domain of the IIEF questionnaire, was not reached. The study was limited in being a small sample size trial with relatively short-term use of pomegranate extracts for treatment of ED; nevertheless, there was a signal for an efficacy of pomegranate extracts to improve mild-to-moderate ED. Further randomized controlled trials that are better powered to assess the efficacy of pomegranate extracts in ED therapy are, therefore, needed. In addition to this clinical trial, studies have examined the efficacy of pomegranate extracts to improve maladies that are typical risk factors for the development and progress of ED, including hypertension and the ability to inhibit serum angiotensin converting enzyme [63]. The management of these well-defined risk factors is an important factor in the overall management of ED.

### 4.3. Benign Prostate Hyperplasia (BPH)

There is currently no completed clinical study evaluating the effect of pomegranate extracts in the treatment of BPH. However, a previous clinical trial provided justification for clinical testing in subjects with BPH [55]. In this study, nitric oxide metabolites were measured to evaluate the level of antioxidant activity in patients treated with pomegranate juice. Compared to baseline, there was a 23% increase in serum nitric oxide metabolites measured in patients serum at 9 months (*P* = 0.0085) with two-thirds of patients assayed having an increase compared to baseline. As described above, the increase in NO levels appears to be a key factor in lowering the tension of smooth muscle of the lower urinary tract via the NO/cGMP pathway and subsequently improving LUTS symptoms. Furthermore, the neoadjuvant POMx pill study [58] demonstrated that POMx reduced 8-OHdG in both benign as well as malignant prostate tissue. The data suggesting decreasing oxidative damage with simultaneous increase in serum NO content support the study of the use of pomegranate extract in order to improve LUTS. Thus, a randomized, double-blind, placebo-controlled pilot study evaluating the efficacy of POMx on BPH-related LUTS is currently under way at our institution.

## 5. Overview of Registered, Ongoing Clinical Trials Evaluating the Effects of Pomegranate Extracts on Urological Diseases

See Table 1.

## 6. Summary

The biological processes of inflammation, hypoxia, and oxidative stress have a crucial function in the natural biology of men's urological diseases including ED, BPH, and PCA. *In vitro* and *in vivo* preclinical experiments provide evidence supporting that pomegranate extracts are able to (i) inhibit proliferation, invasion, metastatic spread, development of castration-resistant PCA growth, and angiogenesis, (ii) modulate inflammatory pathways, and (iii) reduce oxidative stress. Clinical biologic activities of pomegranate have been tested in subjects with PCA and ED. Further randomized, double-blind, controlled trials are under way and will be completed soon.

## Figures and Tables

**Table 1:** 

Trial name	Intervention	Outcome	Project finish date
Effects of plant extracts on semen quality (NCT01357044)	Pomegranate versus placebo	Primary outcome measures: (i) count of motile sperm per ejaculate (time frame: up to two years) (designated as safety issue: no) Secondary outcome measures: (i) morphology: percentage of morphologically abnormal sperm in the ejaculate (time frame: up to two years) (designated as safety issue: no) If we find an increase in the primary outcome measure in data from participants receiving plant extracts and not in those receiving placebo tablets, we wish to further investigate whether sperm morphology also improves when consuming the plant extracts, for example, whether the percentage of abnormal sperm in an ejaculate decreases in the group of participants receiving the active plant extracts	October 2012

Study of POMELLA extract to treat prostate cancer (NCT01100866)	Pomegranate versus placebo	Primary outcome measures: (i) tissue collection and bioanalysis of the specimens collected (time frame: tissue collected on day 31 (after 30 days of study treatment)) (designated as safety issue: no) Prostate tissue are collected at radical prostatectomy. These specimens are used to explore the effects of treatment on the expression of the proliferation markers, enzymes, hormones, receptors, and cell signaling proteins known to influence prostate cancer progression	December 2013

Pomegranate Juice in treating patients with recurrent prostate cancer (NCT00060086)	Pomegranate juice versus placebo	Primary outcome measures: (i) clinical efficacy, in terms of overall response rate, measured by serum prostate-specific antigen (PSA) levels every 3 months (time frame: evaluated every 3 months for 18 months) (designated as safety issue: no)	December 2012

Extension to study of effects of pomegranate extract on rising PSA levels after primary therapy for prostate cancer (NCT00732043)	Pomegranate extract versus placebo	Primary outcome measures: (i) the primary outcome variable will be the mean PSA doubling time at the end of 12, 24, 36, and 48 months (time frame: 48 months) (designated as safety issue: no) Secondary outcome measures: (i) the mean change in PSA doubling time from baseline to end of treatment (time frame: 48 months) (designated as safety issue: no) (ii) response rates in positive and negative PSA doubling times with a clinically significant positive doubling time is defined as >150% of baseline (time frame: 48 months) (designated as safety issue: no) (iii) overall efficacy responses categorized as objective response, progressive disease, and stable disease (time frame: 48 months) (designated as safety issue: no) (iv) measures of tolerability (adverse events) and toxicity (clinical chemistries, etc.) (time frame: 48 months) (designated as safety issue: yes)	January 2015

A pilot study to evaluate the effect of pomegranate juice on semen parameters in healthy male volunteers (NCT01595308)	Pomegranate	Primary outcome measures: (i) change in sperm counts (time frame: baseline and 6 months) (designated as safety issue: no) The primary outcome will be change in sperm counts relative to baseline with and without POM Secondary outcome measures: (i) change in sperm count (time frame: 6 months) (designated as safety issue: no) The secondary outcome will be change in sperm counts relative to baseline with and without POM	May 2012

Open-label extension study of the effects of pomegranate extract on rising PSA after primary therapy for prostate cancer (NCT00731848)	Pomegranate liquid extract	Primary outcome measures: (i) the within-subject difference between the PSA doubling time from the end of double-blind placebo treatment to the end of open-label pomegranate extract treatment (time frame: 12 months) (designated as safety issue: no) Secondary outcome measures: (i) the effect of treatments on response rates for positive PSA doubling times (greater than 150% baseline), for negative posttreatment PSA doubling time (i.e., declining PSA), and for changes in absolute PSA values (time frame: 12 months) (designated as safety issue: no)	January 2015

For more information go to: http://clinicaltrials.gov/.
