# Erratum to “Pomegranate Extracts in the Management of Men's Urologic Health: Scientific Rationale and Preclinical and Clinical Data”

**DOI:** 10.1155/2013/870454

**Published:** 2013-06-10

**Authors:** Nils Kroeger, A. S. Belldegrun, A. J. Pantuck

**Affiliations:** ^1^Department of Urology, Institute of Urologic Oncology, David Geffen School of Medicine at the University of California, 924 Westwood Boulevard, Suite 1050, Los Angeles, CA 90095-7384, USA; ^2^Department of Urology, University Medicine Greifswald, F.-Sauerbruch-Straße, 17489 Greifswald, Germany

The authors' names were incorrectly listed as Kroeger N., Belldegrun A. S., and Pantuck A. J.; the correct format is shown above.

